# Biological properties and roles of a *Trichinella spiralis* inorganic pyrophosphatase in molting and developmental process of intestinal larval stages

**DOI:** 10.1186/s13567-020-00877-8

**Published:** 2021-01-07

**Authors:** Chen Xi Hu, Jie Zeng, Hui Nan Hao, Yang Xiu Yue Xu, Fang Liu, Ruo Dan Liu, Shao Rong Long, Zhong Quan Wang, Jing Cui

**Affiliations:** Department of Parasitology, Medical College, Zhengzhou University, Zhengzhou, 450052 China

**Keywords:** *Trichinella spiralis*, inorganic pyrophosphatase, enzymatic activity, molting, RNAi

## Abstract

Inorganic pyrophosphatase (PPase) participates in energy cycle and plays a vital role in hydrolysis of inorganic pyrophosphate (PPi) into inorganic phosphate (Pi). The aim of this study was to investigate the biological properties of a *Trichinella spiralis* PPase (TsPPase) and its role in larval molting and developmental process. The predicted TsPPase consisted of 367 amino acids with a molecular mass of 41.48 kDa and a pI of 5.76. Amino acid sequence alignment and phylogenetic analysis showed that the TsPPase gene encodes a functional family I soluble PPase with the same characteristics as prokaryotic, plant and animal/fungal soluble PPase. The rTsPPase was expressed and purified, it has the activity to catalyze the hydrolysis of PPi to Pi, and the activity was dependent on Mg^2+^, pH and temperature. The enzymatic activity of rTsPPase was significantly inhibited after its metal binding sites mutation. TsPPase was transcribed and expressed in all *T. spiralis* phases, especially in muscle larvae (ML) and intestinal infective larvae (IIL). Immunofluorescence assay (IFA) revealed that TsPPase was mainly located in cuticle and stichosome. When the ML and IIL were treated with TsPPase-specific siRNA-279, TsPPase expression and enzymatic activity were obviously reduced, the larval molting and development were also impeded. Intestinal IIL as well as AW burden, IIL molting rates from mice infected with siRNA-treated ML were obviously suppressed. The results indicated that rTsPPase possesses the enzymatic activity of native inorganic pyrophosphatase, and TsPPase plays an important role in development and molting process of intestinal *T. spiralis* larval stages.

## Introduction

Trichinellosis is mainly caused by the infection of *Trichinella spiralis* which is a zoonotic tissue-dwelling nematode infecting over 150 kinds of mammals around the world [1]. Human beings suffer from trichinellosis by ingesting raw or semi-raw meat containing *Trichinella* larvae [2–4]. Fifteen trichinellosis outbreaks were documented in China from 2004 to 2009, which consisted of 1387 cases and 4 deaths [5]. Trichinellosis has been a major food-borne zoonosis with economic, social and public health impacts in endemic countries [6–8]. However, it is difficult to eliminate animal *Trichinella* infection due to its broad animal hosts and no practicable anti-*Trichinella* vaccines [9]. Hence, there is an urgent need to find a safe, effective and feasible anti-*Trichinella* vaccine for prevention and control of trichinellosis [10, 11].

After the infected meat is ingested, infectious *T. spiralis* muscle larvae (ML) are released in the stomach from the capsules under the digestion of gastric fluid and develop into intestinal infective larvae (IIL) [12, 13]. Then the IIL invade intestinal epithelium and mature into adult worm (AW) after 4 molts [14, 15]. In 2–3 weeks after infection, each fertilized female deposits about 1500 newborn larvae (NBL), then the NBL migrate, penetrate and encapsulate in host skeletal muscles [16–19].

Inorganic pyrophosphatases (PPases) play a crucial role in energy cycle and the hydrolysis of inorganic pyrophosphate (PPi) into inorganic phosphate (Pi) [20]. This is an exergonic reaction and can be coupled to several unfavorable and energy demanding biochemical transformations such as DNA replication, protein synthesis and lipid metabolism [21]. Rapid hydrolysis of PPi is beneficial to the synthesis of biomacromolecules. PPases are widely distributed in almost all cells and have two major categories, the membrane associated V-H^+^-PPases (H^+^-PPases) and soluble PPases [22]. Because H^+^-PPases are mainly localized in vacuoles such as plant vacuole, bacteria and protists, they are also named vacuolar PPases [23–26]. Soluble PPases have high homology and their active site residues are highly conserved in evolution, they can be divided into three families according to different sequences [22]. Family I contains most of the known soluble PPases, and can be divided into four categories (prokaryotes, fungi, animals and plants) according to their sequence and interspecific distribution. Family II includes *Bacillus subtilis* PPase as well as PPases of four other putative members, two streptococcal and two archeal. There is no similarity between the sequences of the two families [27]. All PPases need the participation of Mg^2+^ for enzyme activity [27].

In recent years, studies on PPases were mainly focused on bacteria and plants, including structure, property, reaction and function of PPases [28–33]. In parasites, the studies on PPases are principally conducted on *Plasmodium falciparum*, *Toxoplasma gondii*, *Schistosoma* and *Ascaris*, and the results showed that PPases are closely related to the parasite development [34]. Therefore, the aim of this study was to investigate the biological properties of a novel PPase of *Trichinella spiralis* (TsPPase Gene ID: 10913356) and its role in the larval molting and developmental process.

## Materials and methods

### Parasites and experimental animals

In this study, *T. spiralis* strain (ISS534) was obtained from domestic swine in central China. The passage was carried out in mice [35]. Female BALB/c mice with six-week-old were purchased from Henan Provincial Experimental Animal Center (Zhengzhou, China).

### Worm collection and protein preparation

In this study, 0.33% pepsin and 1% HCl were used to digest *T. spiralis-*infected mouse muscles at 42 days post-infection (dpi) to obtain the ML [36, 37]. The IIL were acquired from intestine of infected mice. The AW were obtained at 3 and 6 dpi from infected mouse duodenum and jejunum [38]. Female adult worms were cultured in 10% FBS RPMI-1640 complete medium at 37 °C for 48 h, then the NBL were recovered as reported before [39]. The soluble protein and excretory-secretory (ES) proteins of *T. spiralis* in various stages were prepared as before [40–42]. Briefly, the worms were firstly homogenized with tissue grinder for 30 min and the worm fragments were further homogenized with ultrasonication (99 times 3-s cycle, 100 W, 0 °C) The supernatant containing worm crude proteins was recovered following centrifugation at 15,000 *g* for 30 min at 4 °C. To collect worm ES proteins, the worms were washed with sterile saline and then cultured in RPMI-1640 medium at the density of 5000 worms/ml for 18 h at 37 °C in 5% CO_2_. After the media carrying ES proteins were filtered with 0.22 μm membrane, the ES proteins were concentrated using ultrafiltration tubes. The concentrations of these proteins were measured with Bradford method.

### Bioinformatics analysis of TsPPase

Complete TsPPase cDNA sequence was acquired from GenBank (Gene ID: 10913356). The characteristics of TsPPase gene were analyzed using bioanalysis software and websites [43, 44]. The tertiary structure and functional site of TsPPase protein were predicted using PyMOL software and CN3D software. TsPPase amino acid sequences were compared with inorganic pyrophosphatases from other organisms by using Clustal X [13]. The GenBank accession number of inorganic pyrophosphatases from other organisms were as follows: *T. nelsoni* KRX22149.1, *T. patagoniensis* KRY19479.1, *T. britovi* KRY49698.1, *T. murrelli* KRX48666.1, *T. nativa* KRZ58912.1, *Trichinella* sp. T6 KRX75543.1, *Trichinella* sp. T9 KRX66521.1, *Trichinella* sp. T8 KRZ89249.1, *T. papuae* KRZ71476.1, *T. pseudospiralis* KRX90591.1, *T. zimbabwensis* KRZ02917.1, *Wuchereria bancrofti* EJW74547.1, *Brugia malayi* XP_001894848.1, *Ascaris suum* BAC66617.1, *Caenorhabditis elegans* NP_001023073.1, *Ancylostoma duodenale* KIH69146.1, *Necator americanus* XP_013298104.1, *Mus musculus* EDL32138.1 and *Homo sapiens* NP_789845.1. Maximum parsimony method was used to construct phylogenetic tree of the sequences of PPases by MEGA7.0 [45].

### Preparation of rTsPPase and anti-rTsPPase serum

PCR was used to amplify the full-length cDNA of TsPPase by specific primers carrying BamH I and Pst I. Then the PCR products were cloned into pQE-80L. The recombinant plasmid pQE-80L/TsPPase was transferred into *E. coli* BL21, and induced at 37 °C for 6 h using 1.0 mM IPTG [46]. Ni-NTA-Sefinose resin (Sangon Biotech, Shanghai, China) was used to purify rTsPPase protein and SDS-PAGE was used to analyze the rTsPPase. After purification, 20 μg rTsPPase was used to immunize 15 female BALB/c mice, respectively. Three boost immunization were administered as the same rTsPPase dosage at a 14-day interval [47, 48]. Two weeks after the last immunization, 100 μL murine tail blood was collected, and antiserum was isolated [49]. Western blot was used to identify the antigenicity of rTsPPase, anti-rTsPPase IgG antibody titers of antiserum were measured by ELISA with rTsPPase as coating antigen [50].

### Quantitative real-time PCR (qPCR)

Total RNAs of different *T. spiralis* stages (NBL, ML, IIL and AW) were extracted with Trizol reagent (Invitrogen, Carlsbad, CA, USA). The transcription level of TsPPase at various *T. spiralis* stages was investigated by qPCR with specific primers (5′-GATTCGCCCGATTTTGGAGC-3′, 5′-GCGGAATATCATGCCAGGGA-3′) as reported [13]. The internal control gene GAPDH (GenBank: AF452239) of *T. spiralis* was amplified as a positive control and PBS was used to be a negative control. TsPPase transcription level was standardized by deducting the GAPDH transcription level and then calculated by comparative Ct (2^−ΔΔCt^) [13, 51].

### Western blot analysis

Soluble proteins of *T. spiralis* in different stages were separated on SDS-PAGE, then transferred to PVDF membranes (Merck Millipore, Billerica, MA, USA) for 35 min at 18 V, and blocked with 5% skim milk for 2 h at 37 °C. After washing with TBS-0.5% Tween 20 (TBST), the membranes were probed with anti-rTsPPase serum (1:100) at 37 °C for 2 h, subsequently reacted with HRP-anti-mouse IgG conjugate (1:5000; Southern Biotech, Tuscaloosa, AL, USA) at 37 °C for 1 h, mouse antibody against GAPDH (1:1000) was utilized to determine the GAPDH expression as an internal control. Following washing, the membranes were colored with 3, 3′-diaminobenzidine tetrahydrochloride (DAB; Sigma-Aldrich, St. Louis, MO, USA) or using an enhanced chemiluminescent kit (CWBIO, Beijing, China) [52–54].

### Immunofluorescence assay (IFA)

To localize TsPPase in *T. spiralis* worm tissues, IFA was performed with whole worms, and intestinal and muscle sections of infected mice [13, 46]. The tissue was fixed in cold acetone for 20 min; 2-μm intestinal and muscle cross-sections were cut with a microtome-cutting. All the worms and cross-sections were blocked with 1% bovine serum albumin (BSA) and probed by anti-rTsPPase serum diluted at 1:10 at 37 °C for 2 h. After washing with PBS, they were incubated at 37 °C for 1 h with cy3/FITC-anti-mouse IgG conjugate (1:100; Santa Cruz Biotech, Dallas, Texas, USA), finally examined under fluorescence microscopy (Olympus, Tokyo, Japan). The worms and sections were incubated with *T. spiralis*-infected murine serum as the positive control, while they were incubated with normal murine serum as negative control respectively.

### Enzymatic activity assay

Ultraviolet spectrophotometer was used to determine the rTsPPase activity by measuring the rate of release of Pi from PPi through the molybdenum-blue colorimetry [27, 55]. The rTsPPase activity was assayed in a standard reaction mixture which includes 1 mM PPi (Na_4_P_2_O_7_), 5 mM Mg^2+^ and 100 mM Tris–HCl (pH 7.5), the total volume was 200 μL, at 55 °C for 20 min. Adding 10 μL rTsPPase solution into the mixture to start the enzymatic activity assay and to be finished by adding 1 mL 200 mM glycine–HCl (pH 3.0). After that, 125 μL 1% Na_2_MoO_4_ (in 25 mM H_2_SO_4_) and 2.5% SnCl_2_ soluble (in glycerol) were added to the mixture, and incubated for 15 min at 37 °C. The OD values at 690 nm were measured with UV spectrophotometer (Mapada instruments, Shanghai, China). In order to get the optimal condition of the reaction, different temperatures, pH and Mg^2+^ concentrations were experimented. To further verify the rTsPPase activity, the PPases inhibitors (NaF and inosine-5′-diphosphate trisodium salt, IDP) were used to ascertain whether they could effectively inhibit the rTsPPase activity, NaCl was used as the control. The enzyme activity of rTsPPase was defined as μmol Pi/min/mg of protein. In addition, ATP was also used as a substrate for enzyme activity experiments to measure the rTsPPase activity, and the detection method was the same as PPi.

### Enzyme kinetic study

The Michaelis constant (*K*_m_) and maximum velocity value (*V*_max_) were tested by incubating the rTsPPase with increased concentrations of substrates PPi in standard mixture at the optimal conditions.

### Site directed mutagenesis

The rTsPPase played a catalytic role when its metal binding sites combine with divalent metal ions [20], in order to further study its enzyme activity, the site directed mutagenesis was also performed in the present study. The metal binding sites of TsPPase were mutated into amino acids that could not bind with metal ions, the site-specific mutant sequence was chemically synthesized by Sangon Biotech Co., Ltd. (Shanghai, China) and introduced into *Escherichia coli* [56]. The mutant TsPPase (M-TsPPase) was also induced at 37 °C for 6 h using 1.0 mM IPTG, and purified with Ni-NTA-Sefinose resin. Anti-rTsPPase serum and infection serum were used to evaluate the antigenicity of M-TsPPase on Western blotting analysis. Moreover, the enzymatic activity of M-TsPPase was detected by using substrate PPi in the optimal conditions, the rTsPPase was used as the positive control.

### Detection of native TsPPase activity at different stage worms

Various stages of *T. spiralis* worms (NBL, ML, 0.9 h IIL, 10 h IIL, 15 h IIL, 23 h IIL and AW) were obtained and their soluble proteins were prepared. All the soluble proteins were diluted to the same concentration (2.0 μg/μL) and incubated with PPi in the standard reaction mixture under the optimal conditions to detect the native TsPPase activity [27].

### RNA interference experiment

TsPPase-specific siRNA-279 (5′-ACGAUAAAUUCACAUGAUGTT-3′) was designed and synthesized (Sangon Biotech, Shanghai, China). A scrambled sequence (5′-UACAUGCUCGCAAUAAUCATT-3′) was also synthesized as the control siRNA [57]. In order to obtain the optimum interference condition, the ML were transfected with different concentrations of siRNA-279 by electroporation, then cultured in RMPI-1640 medium at 37 °C for different days [58]. Transcription and expression level of TsPPase in siRNA-treated and control larvae were detected by qPCR and Western blot, the level of GAPDH was also determined as an internal standard [54, 59]. The enzyme activity of native TsPPase and larval molting were also investigated after RNAi.

### Detection of native TsPPase activity after RNAi

In order to detect the changes of native TsPPase activity in ML and IIL soluble protein after RNAi, ML and IIL were treated with 4 μM siRNA-279 and cultured for 2 days at 37 °C [54]. Then the worms were obtained and their soluble proteins were prepared. All the soluble proteins were diluted to the same concentration (2.0 μg/μL) and incubated with PPi in the standard reaction mixture at the optimal conditions to detect the native TsPPase enzymatic activity. Untreated worms were used as controls [27, 55].

### Larval molting inhibition assay in vivo

After invasion of the intestine in host, *T. spiralis* needs 4 molts to develop into adult worm, the intestinal larvae are divided into four stages according to the molting time, include L_1_ larvae (0.9 h after infection), L_2_ larvae (10–14 h), L_3_ larvae (15–22 h) and L_4_ larvae (23–30 h) [14, 15, 60, 61]. In order to further verify the role of rTsPPase in the molting and development of *T. spiralis*, three experimental groups were set up to collect 12 h IIL (L_2_ larvae), 24 h IIL (L_4_ larvae) and 3 days AW. One hundred and twenty mice were randomly divided into four groups (30 animals per group), and each group was infected orally with 500 ML treated with NaF, siRNA-279, control-siRNA or PBS, respectively. Ten mice of each group were euthanized at 12 and 24 h, and 3 days after infection. The 12 h IIL, 24 h IIL and 3 days AW were collected and observed with a microscope, the number, length of larvae and molting rate were compared among different groups.

### Statistical analysis

Data of the present study were statistically analyzed by SPSS 21.0. All data were shown as arithmetic mean ± SD (standard deviation). After being tested by Shapiro–Wilk’s test and Levene’s test to check the datum normality and homogeneity, One-way ANOVA and Chi-square test were used to analyze the difference in relative TsPPase mRNA transcription, protein expression, enzyme activity and larval molting rate. Statistical difference level was *P* < 0.05.

## Results

### Bioinformatics analysis of TsPPase

**Figure 1:**
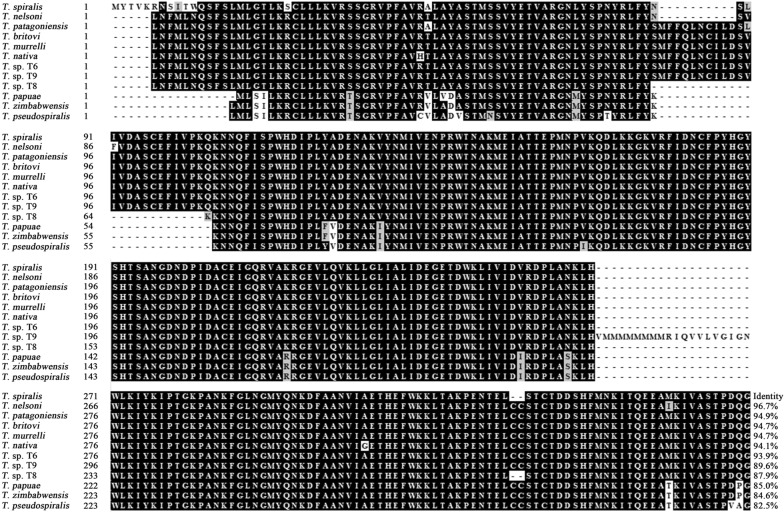
**Sequence alignment of TsPPase gene with other *Trichinella* species.** The TsPPase sequences were analyzed by Bioedit, the sequences have high homology, black shading represents the residues identical to TsPPase. The amino acid sequence had a high identity with inorganic pyrophosphatase of the other* Trichinella* species: *T. nelsoni* (96.7%), *T. patagoniensis* (94.9%), *T. britovi* (94.7%), *T. murrelli* (94.7%), *T. nativa* (94.1%), *Trichinella* sp. T6 (93.9%), *Trichinella* sp. T9 (89.6%), *Trichinella* sp. T8 (87.9%), *T. papuae* (85.0%), *T. zimbabwensis* (84.6%), and *T. pseudospiralis* (82.5%).

Complete TsPPase coding sequence consisted of 1104 bp encoding 367 amino acids (aa), with a 41.48 kDa molecular weight (MW) and 5.76 isoelectric point (pI). There was no signal peptide tested by Signal P 4.1 Server. TsPPase has no transmembrane domain which was detected by TMHMM prediction. The TsPPase homology comparison of inorganic pyrophosphatase compared with those of other species or genotypes of *Trichinella* is shown in Figure 1. The TsPPase amino acid sequence had a high identity with inorganic pyrophosphatase of other *Trichinella* species *T. nelsoni* (96.7%), *T. patagoniensis* (94.9%), *T. britovi* (94.7%), *T. murrelli* (94.7%), *T. nativa* (94.1%), *Trichinella* sp. T6 (93.9%), *Trichinella* sp. T9 (89.6%), *Trichinella* sp. T8 (87.9%), *T. papuae* (85.0%), *T. zimbabwensis* (84.6%), and *T. pseudospiralis* (82.5%).

**Figure 2:**
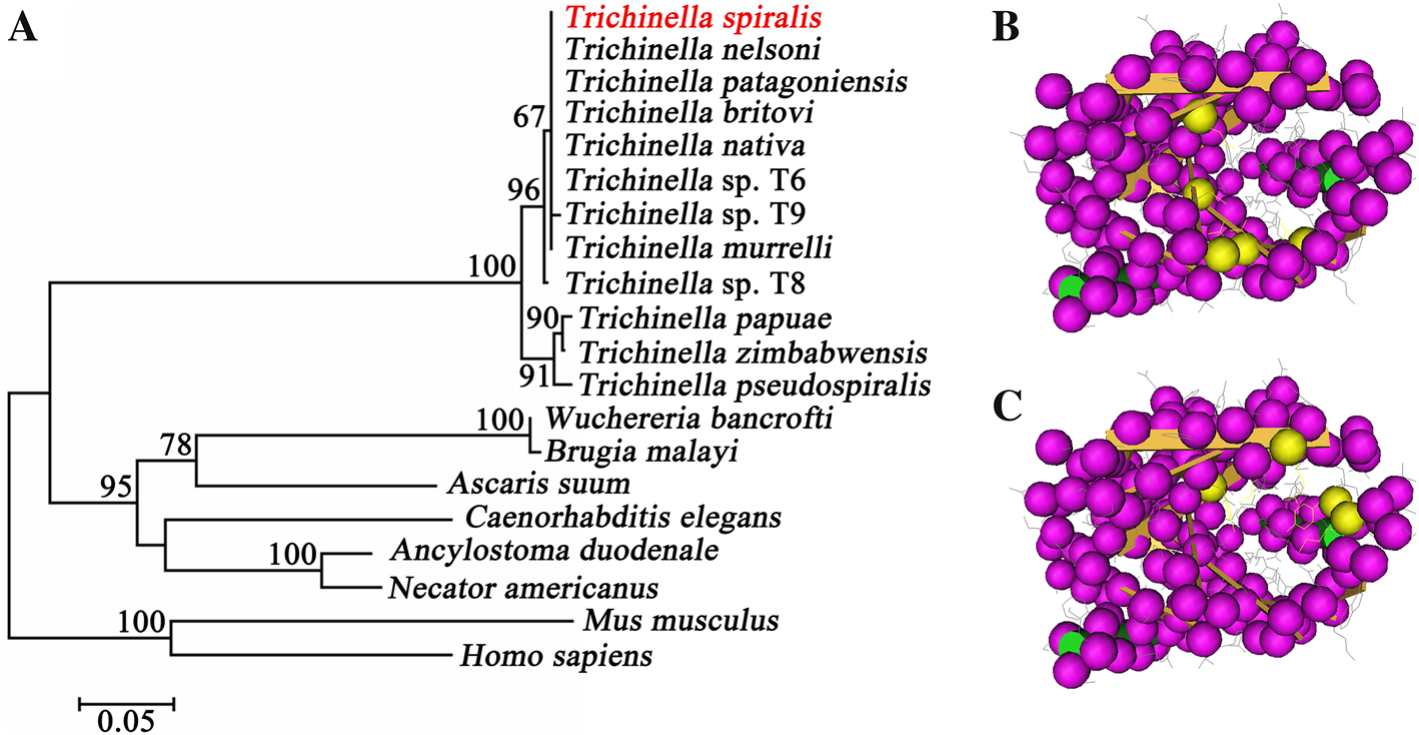
**Phylogenetic tree of inorganic pyrophosphatase of 20 organisms generated with MP method (A) and the predicted 3-dimensional structure of TsPPase protein (B, C).** According to the phylogenetic analysis of inorganic pyrophosphatase, *T. spiralis* has a closer evolutionary relationship with the encapsulated species of *Trichinella* spp. **A** The structure prediction revealed that TsPPase is consisted of 2 α-helices and 12 β-strands, the yellow spherical part represents the metal binding sites (**B**) and the substrate binding site respectively (**C**). The purple spherical part represents the protein backbone, and the small branches represent sidechains.

Phylogenetic analysis of TsPPase with inorganic pyrophosphatase from other *Trichinella* species and nematodes is shown in Figure 2A. The phylogenetic tree shows the monophyletic group of above-mentioned 12 *Trichinella* species/gene types. According to the phylogenetic analysis of inorganic pyrophosphatase, *T. spiralis* has a closer evolutionary relationship with the encapsulated species of the *Trichinella* genus. The structure prediction revealed that TsPPase is consisted by 2 α-helices and 12 β-strands, the yellow spherical parts represent the metal binding sites (Figure 2B) and the substrate binding sites respectively (Figure 2C).

### SDS-PAGE and Western blot analysis of rTsPPase

**Figure 3:**
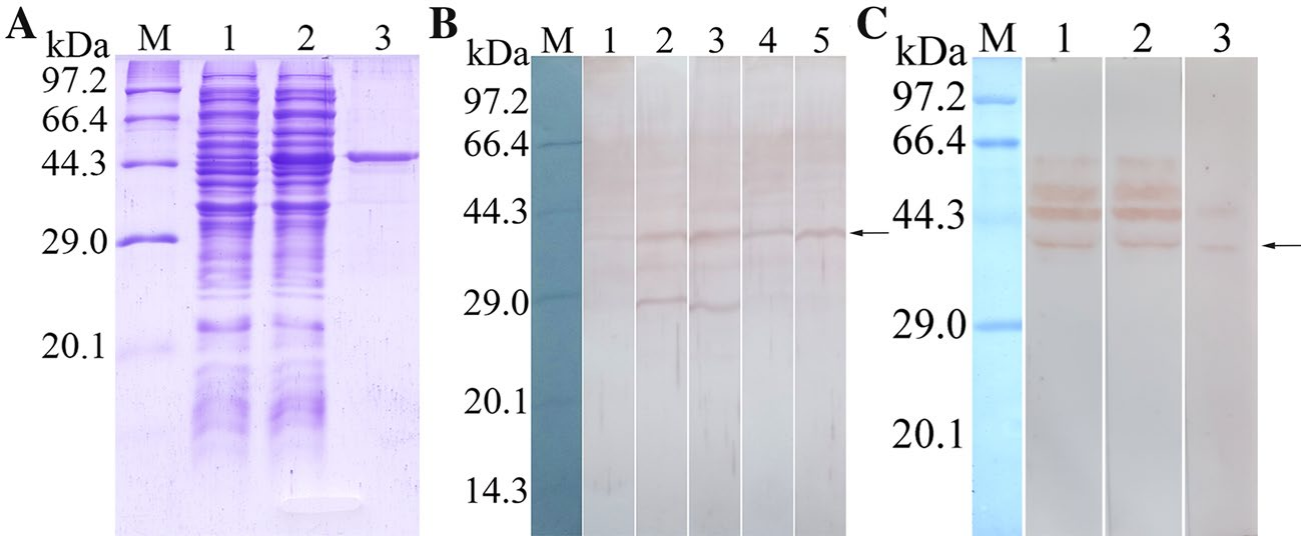
**SDS-PAGE and Western blot analysis of rTsPPase. A** SDS-PAGE of rTsPPase. Lane M: protein marker; lane 1: non-induced recombinant bacterial lysate; lane 2: induced recombinant TsPPase; lane 3: the purified rTsPPase. **B** Western blot revealed native TsPPase in soluble proteins of *T. spiralis* NBL (lane 1), ML (lane 2), IIL (lane 3), 3 days AW (lane 4) and 6 days AW (Lane 5) were probed by anti-rTsPPase serum. **C** Western blot analysis of native TsPPase in ES proteins of ML (lane 1), IIL (lane 2) and AW (lane 3). The arrows showed to the native TsPPase with 41.48 kDa.

The results of SDS-PAGE showed that the *E. coli* BL21 containing pQE-80L/TsPPase expressed an about 44 kDa fusion protein which was a little bigger than the predicted (41.48 kDa). It may be due to the recombinant protein containing a His-tag. The rTsPPase protein exhibited a clear individual band (Figure 3A).

In order to assess the antigenicity of rTsPPase, anti-rTsPPase IgG titers in immunized mice were assayed by ELISA. The results revealed that anti- rTsPPase IgG titers at two weeks after the final immunization reached 1:10^5^, demonstrating that rTsPPase has the immunogenicity (Additional file 1). Western blot analysis was used to detect the soluble and ES proteins of various *T. spiralis* stages (NBL, ML, IIL, 3 days AW and 6 days AW). The results revealed that all soluble and ES proteins of different stages contained native TsPPase, and it could be effectively probed by anti-rTsPPase serum. And the MW (41.48 kDa) of native TsPPase protein was identical with its predicted MW size (41.48 kDa). Another obvious band with 29.0 kDa was also probed in ML and IIL soluble protein by antiserum (Figure 3B). The native TsPPase with 41.0–61.3 kDa in ML and IIL ES proteins were recognized by anti-rTsPPase serum, and native TsPPase with 41.0 and 45.7 kDa was detected in AW ES proteins (Figure 3C).

### qPCR analysis of TsPPase gene transcription at various stages of *T. spiralis*

**Figure 4:**
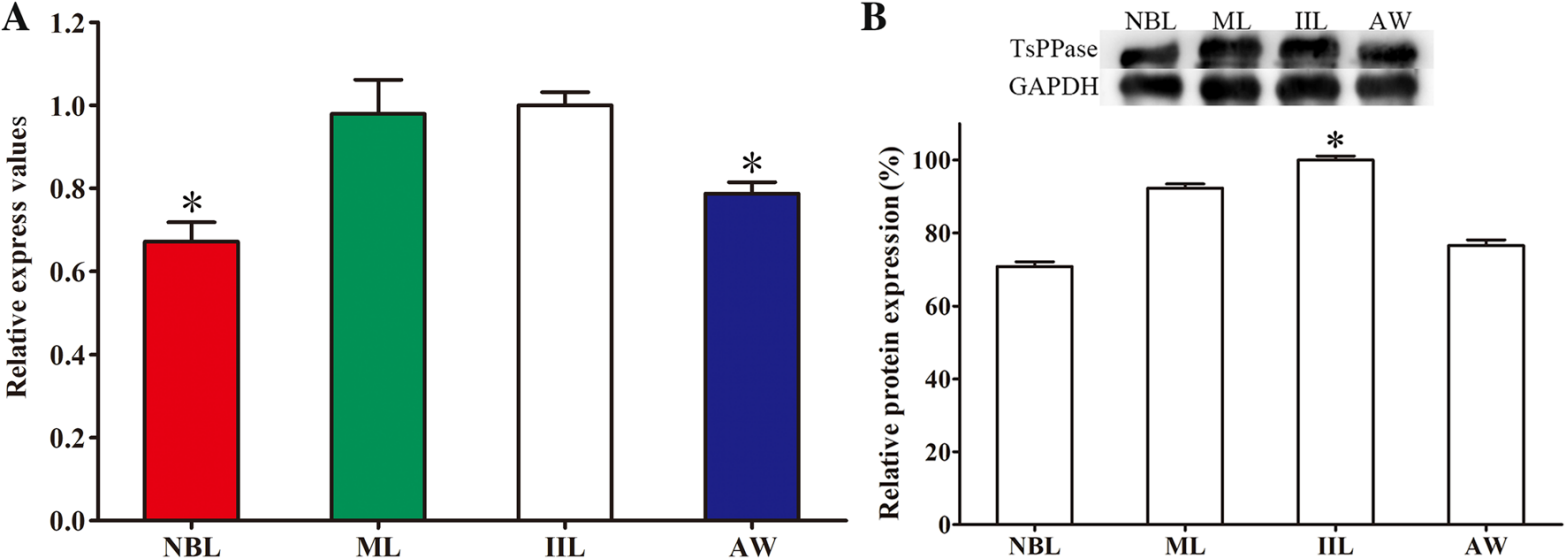
**qPCR and Western blot analysis of TsPPase transcription and expression at various** ***T. spiralis* stages.** The transcription levels were calculated with the Ct (2^−ΔΔCt^) method. *P < 0.05 compared with IIL stage worms (**A**). The expression level at IIL stage was the highest, the difference had statistical significance. *P < 0.05 compared with other stage worms (**B**). GAPDH was utilized as an internal control.

The TsPPase transcription at different phases of *T. spiralis* was detected by qPCR, the result showed that gene was transcribed at all stages of *T. spiralis* (NBL, ML, IIL and AW) (Figure 4). The TsPPase transcription level at IIL stage was higher than that of NBL and AW (*F* = 27.776, *P* < 0.05). The difference of transcription level between IIL and ML stages had no statistical significance (*P* > 0.05) (Figure 4A).

### Western blot analysis of TsPPase expression at different worm stages

The expression level of native TsPPase protein at diverse phases of *T. spiralis* was detected on Western blot. It revealed that the TsPPase protein was expressed at all stages (NBL, ML, IIL and AW). The TsPPase protein expression level in IIL stage was obviously higher than that of other stages and the difference had statistical significance (*χ*^2^_NBL_ = 30.521, *P* < 0.0001, *χ*^2^_ML_ = 4.714, *P* < 0.05, *χ*^2^_AW_ = 22.650, *P* < 0.0001) (Figure 4B). The TsPPase protein expression level was consistent with that of TsPPase mRNA expression level measured by qPCR.

### Expression and localization of TsPPase at diverse *T. spiralis* stage by IFA

**Figure 5:**
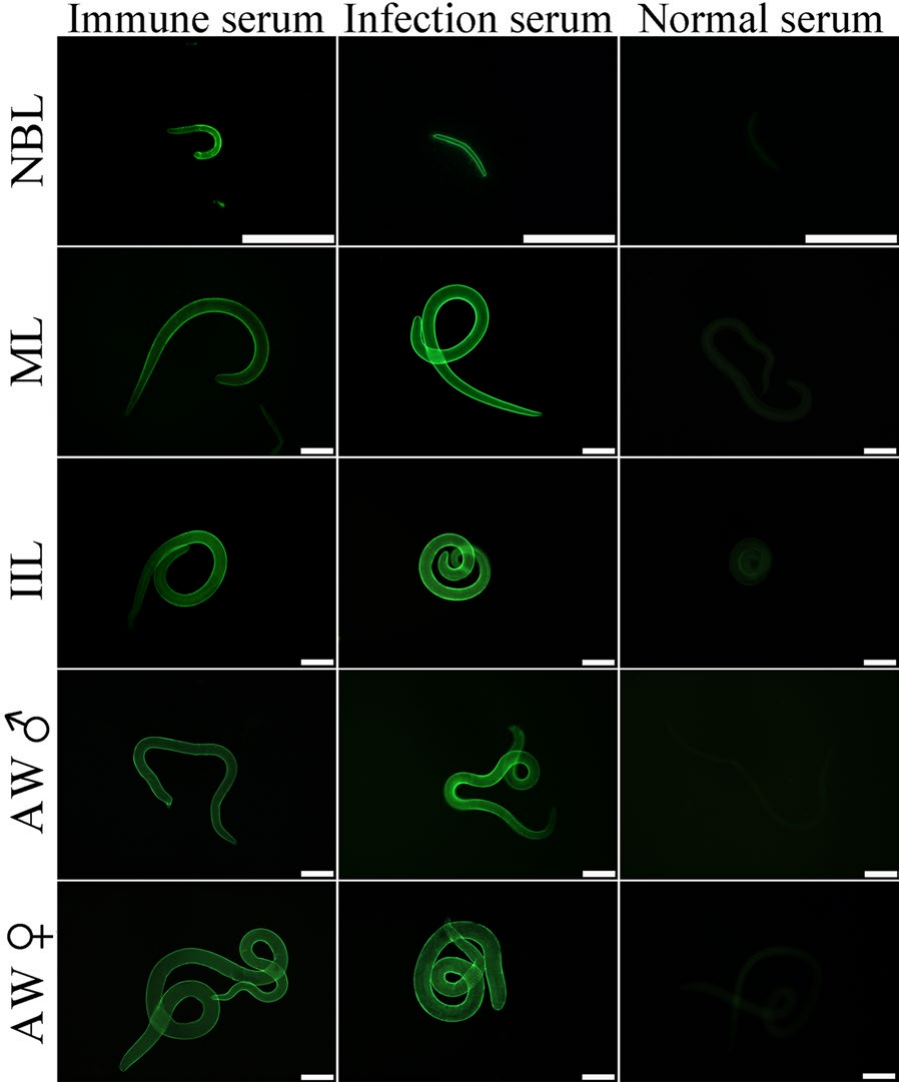
**Immunolocalization of TsPPase in *T. spiralis*** **various stages by IFA with anti-r TsPPase serum.** Fluorescence staining was found at the epidermis of ML, IIL, male AW, female AW and NBL by using anti-rTsPPase serum. The worms recognized by infection serum as a positive control, and normal serum as the negative control. Scale bar: 100 μm.

**Figure 6:**
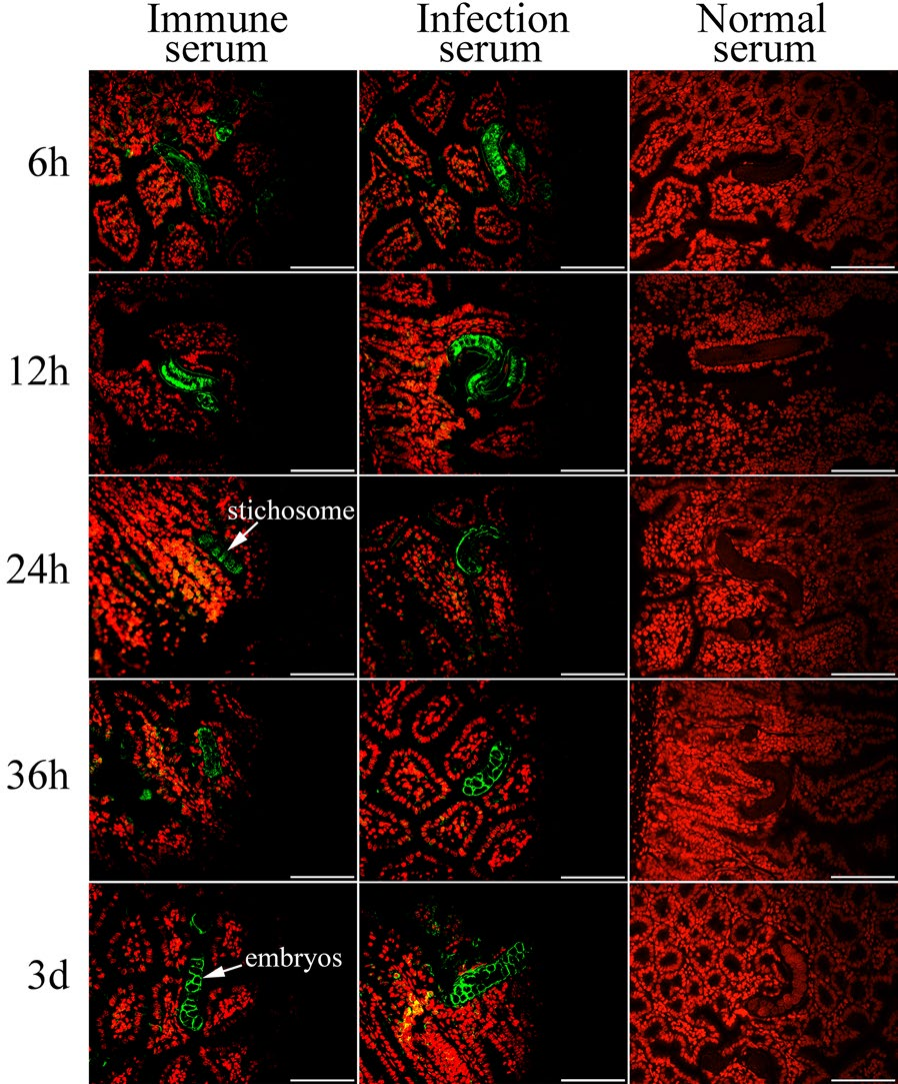
**IFA of *T. spiralis* in infected mouse intestinal tissues.** Immuno-fluorescence staining was observed at all *T. spiralis* intestinal stages, mainly at the stichosome and around the embryos of this nematode. Intestinal tissue sections recognized by infection serum as a positive control, and normal serum as the negative control. The nuclei of intestinal cells were stained red by propidium iodide (PI). Scale bar: 100 μm.

The expression and localization of native TsPPase at different *T. spiralis* stages were determined by IFA. The results of IFA with entire worms showed that positive fluorescence staining was detected at the epidermis of all stage worms (Figure 5), indicating that native TsPPase was expressed at the epidermis of all stage worms. IFA was also used to detect the expression and location of native TsPPase at diverse *T. spiralis* stages in infected murine intestinal and muscle tissues. The results revealed that immuno-fluorescence staining was observed at *T. spiralis* intestinal stages, mainly at the stichosome and around the embryo of this nematode (Figure 6). When *T. spiralis*-infected murine muscle tissue sections were used, immunostaining was observed at muscle larvae by IFA using anti-rTsPPase serum, it was primarily localized at stichosome of the larvae (Additional file 2).

### Determination of the rTsPPase enzyme activity

**Figure 7:**
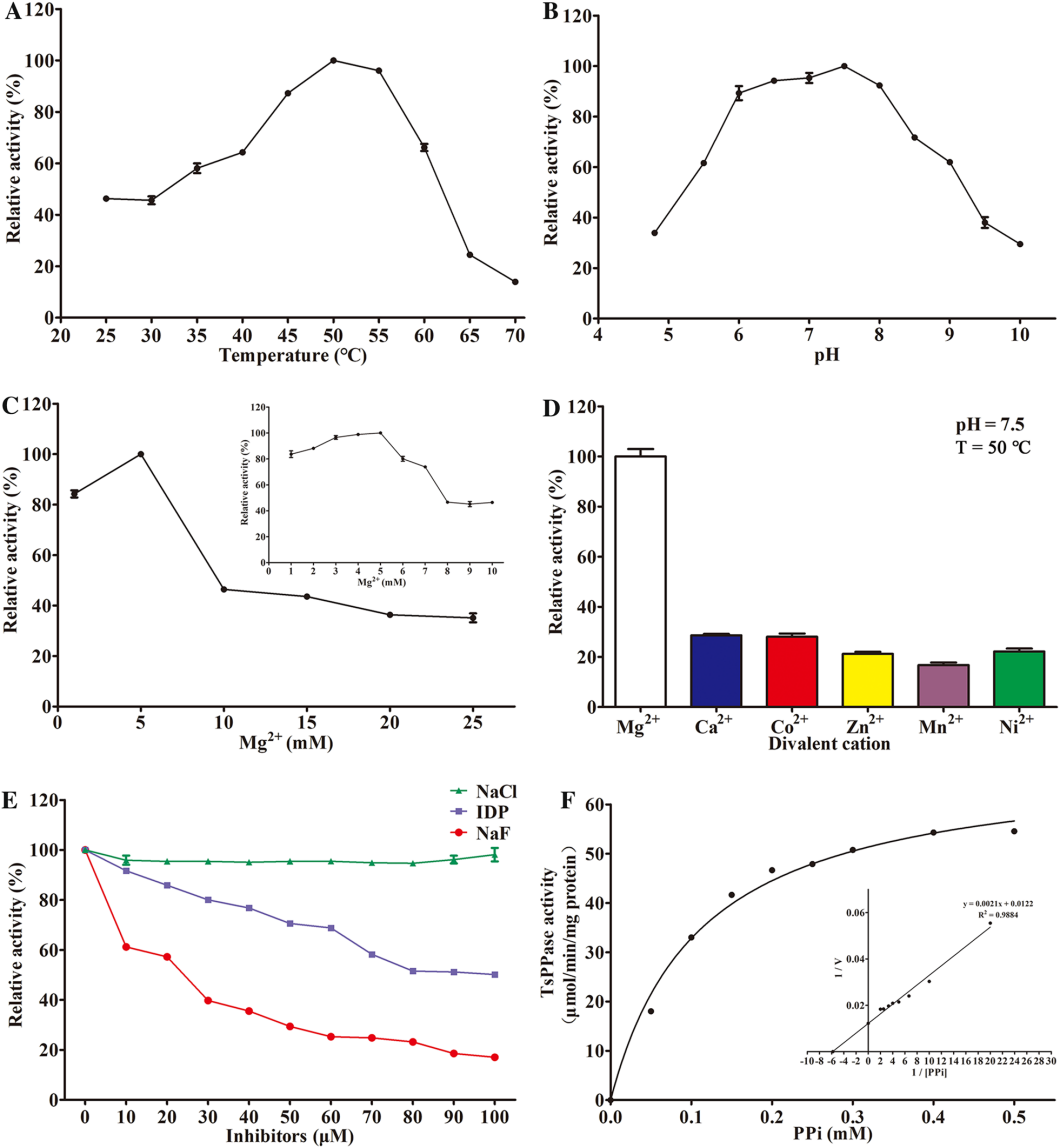
**Enzymatic characteristics of rTsPPase (PPi as substrate). A** Enzymatic activity at temperature 25–70 °C, the optimal temperature was 50 °C. **B** Optimum pH at 50 °C, and the optimum pH was 7.5. **C** Enzymatic activity at various Mg^2+^ concentrations (1–25 mM) at 50 °C and pH 7.5. The highest activity was observed at 5 mM Mg^2+^. **D** Different divalent cation effects on the enzyme activity were compared, the result showed that the ability of Mg^2+^ was the strongest. **E** The enzymatic activity of rTsPPase was obviously inhibited by the inhibitors NaF and IDP with a dose-dependent mode. **F** Michaelis–Menten curve and Lineweaver–Burk Plot at pH 7.5, 50 °C and 5 mM Mg^2+^. The *K*_m_ value was deduced to 170 μM due to the X axis intercept which represent − 1/*K*_m_.

**Figure 8:**
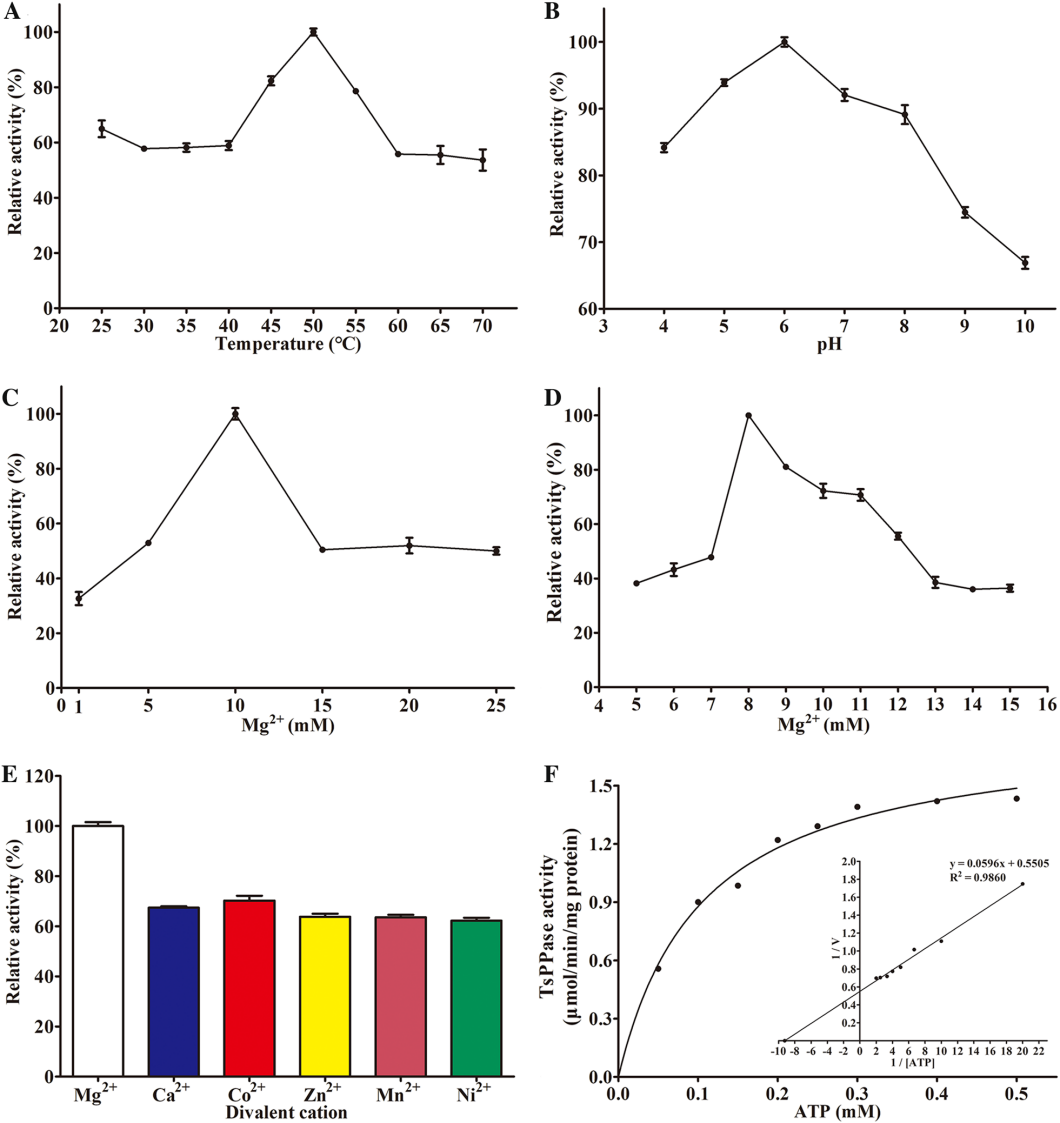
**Enzymatic characteristics of rTsPPase (ATP as substrate). A** Enzymatic activity at temperature 25–70 °C, the best temperature was 50 °C. **B** Optimum pH at 50 °C, and the optimum pH was 6.0. **C**, **D** Enzymatic activity at various Mg^2+^ concentrations (1–25 mM) at 50 °C and pH 6.0. The best activity was observed at 8 mM Mg^2+^. **E** Different divalent cation effects on the enzyme activity were compared, the result showed that the ability of Mg^2+^ was the strongest. **F** Michaelis–Menten curve and Lineweaver–Burk Plot at pH 6.0, 50 °C and 8 mM Mg^2+^. The *K*_m_ value was deduced to 108 μM due to the X axis intercept which represent − 1/*K*_m_.

In order to obtain the optimal condition of the enzymatic reaction, various temperatures, pH and divalent cations were tested in reaction mixture. The absorbance value of reaction mixture was detected by ultraviolet spectrophotometer at 690 nm. Finally, the optimal condition of reaction was gained when the substrate was PPi, the best temperature, pH and Mg^2+^ concentrations were 50 °C, 7.5 and 5 mM, respectively (Figure 7A–C). The effects of different divalent cations on the rTsPPase enzyme activity were also compared, the result showed that the Mg^2+^ ability for rTsPPase catalysis was the strongest (Figure 7D). NaF, an anion, is the potent inhibitor of PPases and was able to inhibit the rTsPPase activity in a dose-dependent manner, IDP also could inhibit the activity of rTsPPase, but the inhibitory effect was not as strong as NaF. And the NaCl had no inhibitory effect (Figure 7E). Furthermore, the PPi dependence of the maximum hydrolytic velocity (*V*_max_) of rTsPPase was shown to be 81.96 μmol Pi/min/mg of protein with a Michaelis constant (*K*_m_) value of 170 μM (Figure 7F). When ATP was used as substrate, the best temperature, pH and Mg^2+^ concentrations were 50 °C, 6.0 and 8 mM, respectively. The ATP dependence of the maximum hydrolytic velocity (*V*_max_) of rTsPPase was shown to be 1.816 μmol Pi/min/mg of protein with a Michaelis constant (*K*_m_) value of 108 μM (Figure 8).

### SDS-PAGE and Western blot analysis of M-TsPPase

**Figure 9:**
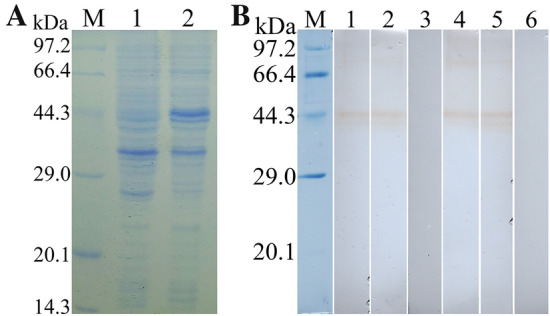
**SDS-PAGE (A) and Western blot (B) analysis of M-TsPPase. A** SDS-PAGE of M-TsPPase expressed by *E. coli* system. M: protein marker; lane 1: non-induced recombinant bacterial lysate; lane 2: induced recombinant pQE-80L/M-TsPPase. **B** Western blot analysis of M-TsPPase, the result showed that the M-TsPPase was probed by anti-rTsPPase serum (lane 1) and murine infection serum (lane 2), but not be recognized by normal serum (lane 3). The rTsPPase was incubated with anti-rTsPPase serum (lane 4), infection serum (lane 5) and normal serum (lane 6).

The results of SDS-PAGE showed that the *E. coli* system expressed an about 44 kDa fusion protein and the MW of M-TsPPase was the same as that of rTsPPase (Figure 9A). After being purified, the Western blot results revealed that the M-TsPPase was also recognized by anti-rTsPPase serum and murine infection serum (Figure 9B). The results demonstrated that the mutation of TsPPase metal binding sites did not affect its antigenic epitopes, the M-TsPPase also had good antigenicity.

### The enzymatic activity of M-TsPPase

**Figure 10:**
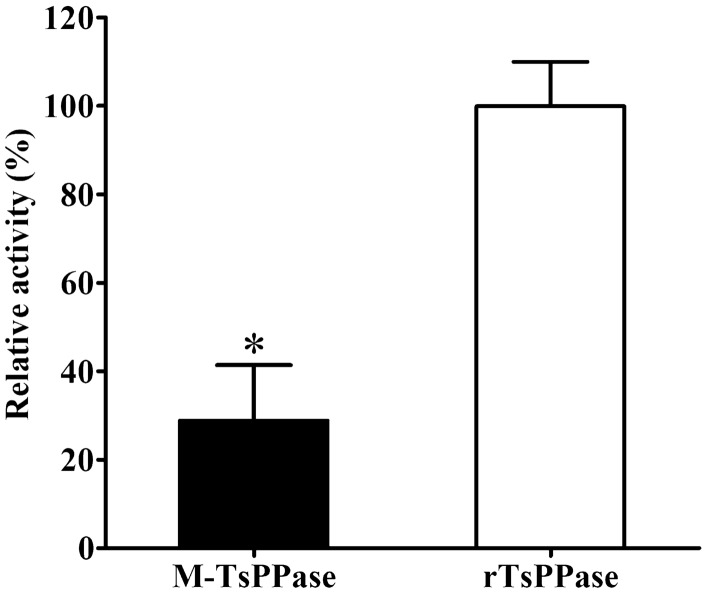
**The enzymatic activity of M-TsPPase.** The enzymatic activity of M-TsPPase was detected by using substrate PPi in the optimal conditions (pH 7.5, 50 °C and 5 mM Mg^2+^), the rTsPPase was used as the control. *P < 0.05 compared with the activity of rTsPPase.

The enzymatic activity of M-TsPPase was determined. The relative activity of rTsPPase was taken as 100%, the result showed that the M-TsPPase only exhibited 24.33% enzymatic activity, the enzyme activity was inhibited by 75.67% after mutation (Figure 10). The difference of TsPPase enzymatic activity before and after mutation was statistically significant (*χ*^2^ = 122.581, *P* < 0.05). The results revealed that the activity was obviously inhibited after the metal binding sites mutation.

### The enzymatic activity of native TsPPase in various stage worms

**Figure 11:**
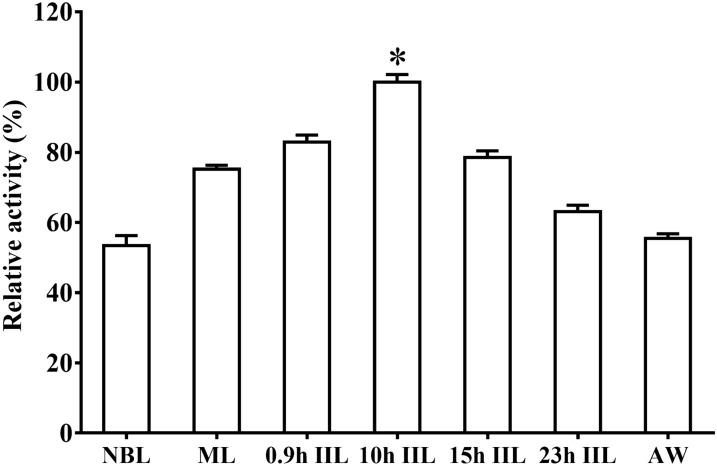
**The enzymatic activity of native TsPPase at different stages of** ***T. spiralis*** **.** All worm soluble somatic proteins were diluted to same concentration and incubated with PPi in standard reaction mixture at the best conditions to detect native TsPPase activity. The TsPPase of 10 h IIL stage showed the highest enzymatic activity. *P < 0.05 compared with other stages.

The activity of native TsPPase in different worm stages was determined, the result revealed that the TsPPase of 10 h IIL stage showed the highest enzymatic activity, and the difference was statistically significant (*χ*^2^_NBL_ = 61.438, *χ*^2^_ML_ = 28.571, *χ*^2^_0.9h_ = 18.579, *χ*^2^_15h_ = 24.719, *χ*^2^_23h_ = 45.399, *χ*^2^_AW_ = 58.065, *P* < 0.05). The activity of native TsPPase at IIL stage were obviously higher than other stages (NBL, ML and AW) (Figure 11), indicating that TsPPase mainly played a role in intestinal phase worms, it might participate in the molting and development of *T. spiralis* larvae.

### TsPPase expression level in siRNA treated ML

**Figure 12:**
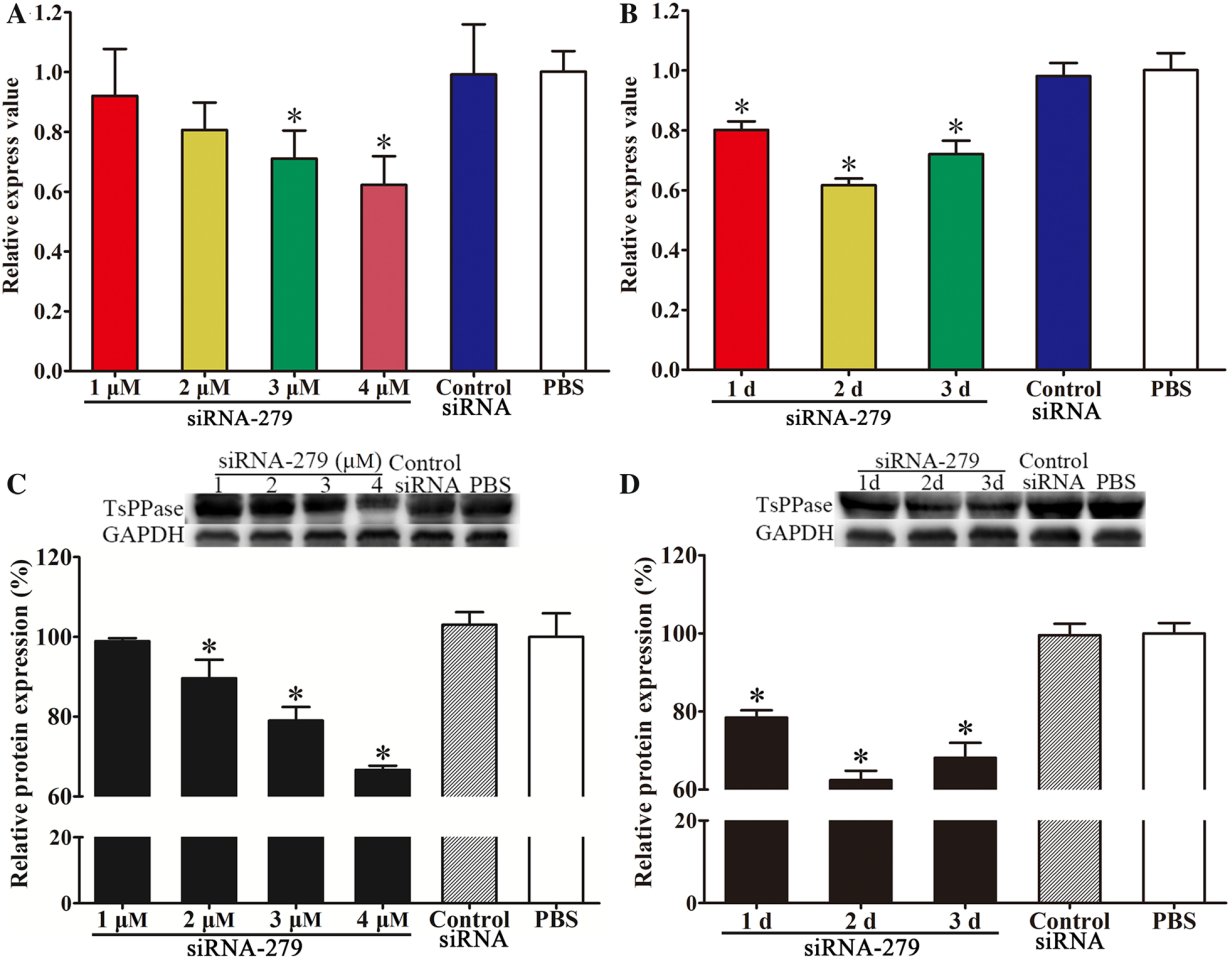
**Reduction of TsPPase transcription and expression in siRNA treated ML. A** The transcription level of TsPPase 1 day after electroporation with different concentrations of siRNA-279. **B** The transcription level of TsPPase at different days after treatment with 4 μM siRNA-279. Western blot was used to detect the TsPPase protein expression level in soluble protein of *T. spiralis* ML treated with different siRNA-279 concentrations (**C**) and various culture days (**D**). *P < 0.05 compared to control siRNA and PBS group.

Different concentrations of siRNA-279 (1, 2, 3 and 4 μM) were transfected into ML by electroporation for 1 day, then qPCR was used to detect the transcription level of TsPPase. The results showed that the ML electroporated using 3 and 4 μM of siRNA-279 exhibited 28.95 and 37.76% reduction of TsPPase transcription level, which was significantly lower than that of PBS group (*F* = 5.194, *P* < 0.05) (Figure 12A). At 1, 2 and 3 days following electroporation using 4 μM of siRNA-279, the transcription level reduced by 19.83, 38.37 and 27.94%, respectively (*F* = 49.025, *P* < 0.05) (Figure 12B). There was no statistical difference between the control siRNA group and PBS group (*P* > 0.05).

Compared to the PBS group, the TsPPase expression level was inhibited by different concentrations of siRNA-279, and there was a 33.34% reduction in 4 μM siRNA-279 treated larvae (*χ*^2^ = 39.521, *P* < 0.05) (Figure 12C). Then the 4 μM siRNA-279 treated worms were cultured in RMPI-1640 for 1, 2 and 3 days, the TsPPase expression level was significantly inhibited with a 37.55% reduction at 2 days following cultivation compared with the PBS group (*χ*^2^ = 46.914, *P* < 0.05) (Figure 12D).

### Determination of native TsPPase enzymatic activity in siRNA treated larvae

**Figure 13:**
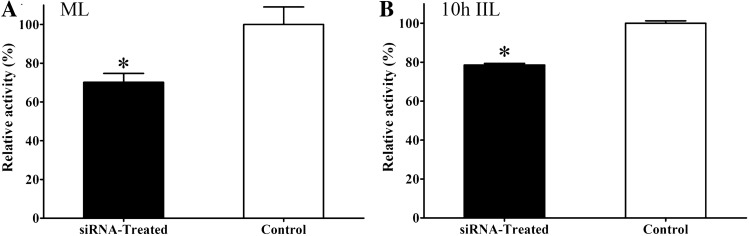
**Inhibition of native TsPPase enzymatic activity in ML (A) and IIL (B) by siRNA-279.** *P < 0.05 compared to the PBS control group (Untreated worms).

Compared with the PBS control group, the enzymatic activity of TsPPase in siRNA treated ML was reduced by 29.83% (*χ*^2^ = 35.294, *P* < 0.05) (Figure 13A). The TsPPase enzymatic activity in siRNA-treated 10 h IIL was also reduced by 21.41% (*χ*^2^ = 24.719, *P* < 0.05) (Figure 13B).

### Suppression of NaF and siRNA-279 on larval molting and development in vivo

**Figure 14:**
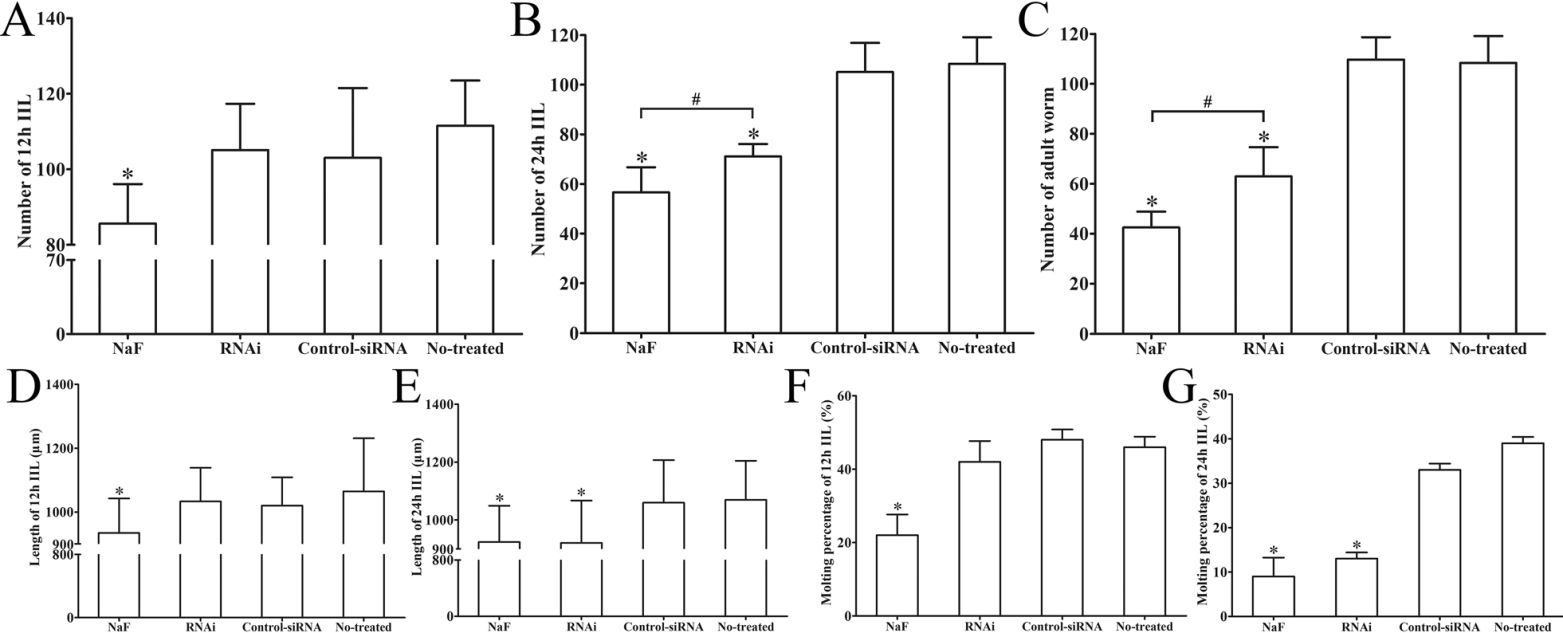
**Suppression of siRNA-279 on larval molting and development.** Comparison of the numbers of 12 h IIL (**A**), 24 h IIL (**B**) and 3 days AW (**C**) collected from intestine of mice infected with siRNA-279 treated ML. Comparison of the length of 12 and 24 h IIL in various groups (**D, E**). The larval molting rate of 12 h IIL (**F**) and 24 h IIL (**G**) were inhibited by NaF and RNAi. *P < 0.05 compared to the PBS group. ^#^*P* < 0.05 between NaF and RNAi groups.

**Figure 15:**
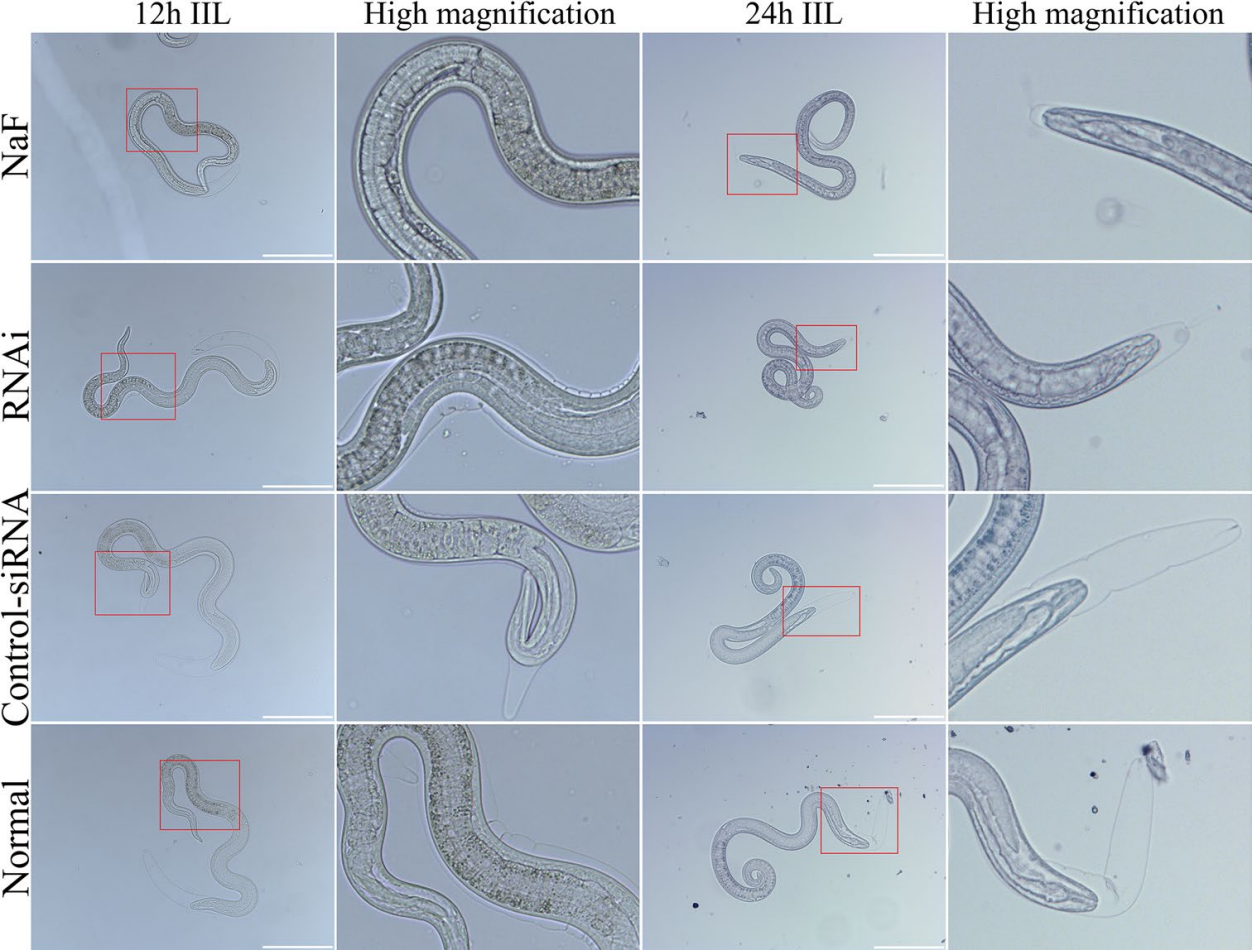
**The larval molting was inhibited by siRNA-279 in IIL stage.** In the 12 h IIL stage, the larval molting was obviously suppressed by inhibitor NaF, no obvious separation between the old and the new cuticle was observed except the larval tail end*,* while the separation of new and old cuticles of 12 h IIL in the other three groups was clearly observed. In the 24 h IIL stage, the obvious suppression of larval molting was also observed in siRNA group due to the successful inhibition of TsPPase expression and activity. The area in the red box was enlarged for observation.

The results of the in vivo molting assay showed that NaF and siRNA-279 had the ability to inhibit the molting and development of *T. spiralis*. Compared with the PBS group, the mice infected with NaF-treated ML exhibited a 23.20, 47.79 and 60.79% reduction of intestinal worms at 12, 24 h and 3 dpi. Whereas the RNAi group exhibited a 32.30 and 42.20% reduction of 24 h IIL and 3 days AW compared to the control-siRNA group (Figure 14A–C). The length of 12 and 24 h IIL in NaF group were significantly shorter than that of the PBS group (*F*_12h_ = 10.673, *F*_24h_ = 9.776, *P* < 0.0001), whereas only 24 h IIL in RNAi group was evidently shorter than that in control-siRNA group (*P* < 0.01) (Figure 14D, E). More importantly, molting of the NaF- and siRNA 279-treated intestinal larvae was restrained (Figure 15). In the NaF-treated group, the 12 and 24 h IIL molting rate were 22 and 10%, significantly lower than 46 and 38% of the PBS group (*χ*^2^_12h_ = 10.176, *χ*^2^_24h_ = 11.080, *P* < 0.01). The siRNA-279 treated 24 h IIL exhibited a 14% molting rate, significantly lower than 34% of control-siRNA group (*χ*^2^_24h_ = 5.482, *P* < 0.05) (Figure 14F, G). All the results revealed that the TsPPase played an important role in *T. spiralis* larval molting and development in hosts.

## Discussion

Inorganic pyrophosphatase (PPase) is a kind of enzymes with catalytic activity, which mainly plays a role in the synthesis of biological macromolecules in life [21]. The main function of PPase is catalytic phosphorylation, PPase can play a catalytic role when its metal binding sites are combined with divalent metal ions [20]. In the process of the reaction, PPase can catalyze the hydrolysis of substrate inorganic pyrophosphate (PPi) into two molecules of phosphate (Pi), thus controlling the concentration of pyrophosphate in cells and regulating the intracellular water-salt balance. In addition, PPase can also catalyze the degradation of ATP to provide energy for life activities [62, 63]. So far, studies on PPase are mainly focalized on tumors and plants, and few studies on parasite PPases are reported in the literatures [36, 64, 65].

Previous studies indicated that inorganic pyrophosphatase (PPase) played an important role in parasite survival in the host, such as in the life cycle of *Plasmodium* and *Toxoplasma gondii*, PPase played an important role in the regulation of growth and cell division [66]. In the study of *Leishmania*, the results revealed that the PPase was involved in phosphate metabolism and provided energy source for the life activities of *Leishmania* [67, 68]. At present, the researches on PPase in roundworm were mainly focused on *Ascaris suum*, the results indicated that the PPase not only played a key role in larval survival in host, but also played a crucial role in larval molting and development, recombinant PPase had the potential to protect the host from infection [27, 34]. The results indicated that the inorganic pyrophosphatase played a very important role in the life cycle of the nematode, and it was mainly involved in larval development. Therefore, the role of PPase in the development of *Trichinella spiralis* was ascertained in this study.

In the present study, a novel *T. spiralis* inorganic pyrophosphatase (TsPPase) was expressed and purified, the mice were immunized with recombinant TsPPase (rTsPPase) and the polyclonal anti-rTsPPase antibodies were prepared. Bioinformatics analysis showed that the TsPPase had no signal peptide and transmembrane domain, the amino acid sequence of TsPPase had a high identity with that of other *Trichinella* species. The phylogenetic tree revealed that the TsPPase had a closer evolutionary relationship with the encapsulated *Trichinella* species. qPCR and Western blotting revealed that the TsPPase was expressed at every life cycle stage of *T. spiralis*, the TsPPase protein was expressed at all stages and it was a secretory protein of this nematode, the TsPPase expression level in IIL stage was significantly higher than other worm stages, the results indicated the TsPPase might play an important role in IIL stage [13, 27]. On Western blotting an obvious band with approximate 29.0 kDa was probed in ML and IIL soluble protein by anti-rTsPPase serum, suggesting that there were other proteins with the same epitopes as TsPPase in ML and IIL soluble protein [43]. Western blotting also showed that several native TsPPase bands in ML, IIL and AW ES proteins were recognized by anti-rTsPPase serum, likely because TsPPase protein might have different isoforms, this protein might be processed by post-translational processing and modification, or TsPPase is a member of the *Trichinella* PPase family that possesses the same antigenic epitopes [48, 69]. IFA was used to localize the TsPPase in *T. spiralis*, and the results showed that positive fluorescence staining was detected at the epidermis of all stage worms, and especially localized at the stichosome and around the embryos. The results suggested that the TsPPase was a secretory protein which was likely derived from cuticle/ES protein of this nematode [40, 48].

Enzymatic activity assay was performed and it revealed that the rTsPPase had natural catalytic activity with the specific substrate PPi, and determined a *K*_m_ value 170 μM at an optimum condition. The rTsPPase also played a catalytic role in promoting ATP degradation with the substrate ATP, and the *K*_m_ value was 108 μM at the optimum condition. The catalytic activity of rTsPPase was inhibited by NaF when PPi and ATP were used as substrates, the reason is that F^−^ can replace metal ions such as Mg^2+^ to bind to the metal binding sites of rTsPPase, thus inhibiting the enzyme activity [27, 34]. After mutating the metal binding sites of TsPPase, the M-TsPPase activity was significantly inhibited due to the decrease of its ability to bind metal ions, indicating that metal ions were necessary for TsPPase enzymatic activity [27]. The activity of native TsPPase in various *T. spiralis* stages was also detected at the optimum condition, the native TsPPase activity in IIL stage was significant higher than other stages, further suggesting that TsPPase might play a crucial role in IIL molting and growth and the result was consistent with the results of qPCR and Western blot [16].

In previous researches on parasites, the RNA interference technique was widely used [54]. In order to further validate the function of rTsPPase involved in larval molting and development, RNAi was used in this study. The TsPPase transcription level was obviously inhibited by siRNA-279, the TsPPase protein expression level was suppressed by 37.55% when *T. spiralis* worms were treated with 4 μM siRNA-279 for 2 days. And the catalytic activity of native TsPPase in ML and IIL were also inhibited by 29.83% and 21.41%, respectively. In the in vivo molting inhibition assay, the mice were orally infected using ML treated with NaF, siRNA-279, control-siRNA or PBS, respectively. The results showed that larval molting and development was restrained when the TsPPase activity was inhibited and silenced by NaF and siRNA-279. The length of 12 and 24 h IIL in NaF group were significantly shorter than that of the PBS group, it is possibly because the larvae treated with NaF and siRNA-279 were incapable to further molt and develope in host intestine. Furthermore, the 24 h IIL in RNAi group was shorter than that of control siRNA group, and the larval molting was also inhibited. Because the larva molting was inhibited and could not develop into adult worms, the number of recovered 3 days AW in NaF and RNAi groups was significantly lower than that of control-siRNA and PBS groups [70]. However, the results showed that the siRNA inhibitory effect was not as strong as that of NaF. The results demonstrated that TsPPase played a crucial role in the molting and development of enteral *T. spiralis* larval stages [27, 34].

In conclusion, the role of TsPPase in *T. spiralis* larval molting and development was identified in this study, TsPPase was transcribed and expressed at all the life cycle phases of *T. spiralis*, and highly expressed in intestinal larval stage, mainly located in the cuticle and stichosome of ML and IIL, it was also localized around the embryos of female adult worms. The rTsPPase had the native catalytic activity to catalyze the degradation of PPi and ATP, its catalytic activity could be inhibited by specific NaF and IDP. When the transcription and expression of TsPPase was suppressed by the inhibitors and RNAi, the larval molting and development were hindered, resulting in the failure of larval development into adulthood. The results of this study indicated that the TsPPase plays a crucial role in the *T. spiralis* molting and development, and it could be a promising candidate vaccine target molecular against trichinellosis.

## Supplementary information

**Additional file 1.** **Serum anti-rTsPPase IgG titers measured by ELISA with rTsPPase as coating antigen.** Forty normal murine serum samples (1:100 dilutions) were measured as negative controls. The cut-off value (0.241) was showed as a dotted line.

**Additional file 2.** **IFA of T. spiralis-infected mouse muscle tissue cross sections.** Immunostaining was observed at muscle larvae of *T. spiralis*-infected murine muscle tissue section by IFA using anti-rTsPPase serum, it was primarily localized at stichosome of the larvae. Muscle sections recognized by infection serum as a positive control, and normal serum as the negative control. The nuclei of muscle cells were stained blue by DAPI. Scale bar: 100 μm.

## Data Availability

Not applicable.

## Abbreviations

AW: adult worms
ELISA: enzyme-linked immunosorbent assay
ES: excretory-secretory
HRP: horseradish peroxidase
IFA: immunofluorescence assay
IDP: inosine-5′-diphosphate trisodium salt
IIL: intestinal infective larvae
IPTG: isopropyl β-d-1-thiogalactopyranoside
ML: muscle larvae
M-TsPPase: mutant *T. spiralis* inorganic pyrophosphatase
NBL: newborn larvae
NC: nitrocellulose
OD: optical density
PBS: phosphate-buffered saline
PPi: inorganic pyrophosphate
Pi: **i**norganic phosphate
PPase: inorganic pyrophosphatase
rTsPPase: recombinant *T. spiralis* inorganic pyrophosphatase
SDS-PAGE: sodium dodecyl sulfate-polyacrylamide gel electrophoresis
TBST: tris-buffered saline containing Tween
TsPPase: *T. spiralis* Inorganic pyrophosphatase
