# Supplementary material for: Biological properties and roles of a Trichinella spiralis inorganic pyrophosphatase in molting and developmental process of intestinal larval stages
Source: Vet Res. 2021 Jan 7;52:6. doi: 10.1186/s13567-020-00877-8 (PMC7791673; doi:10.1186/s13567-020-00877-8)
Supplement: Supplementary file 1 — Additional file 1. Serum anti-rTsPPase IgG titers measured by ELISA with rTsPPase as coating antigen. Forty normal murine serum samples (1:100 dilutions) were measured as negative controls. The cut-off value (0.241) was showed as a dotted line. [file 13567_2020_877_MOESM1_ESM.docx]

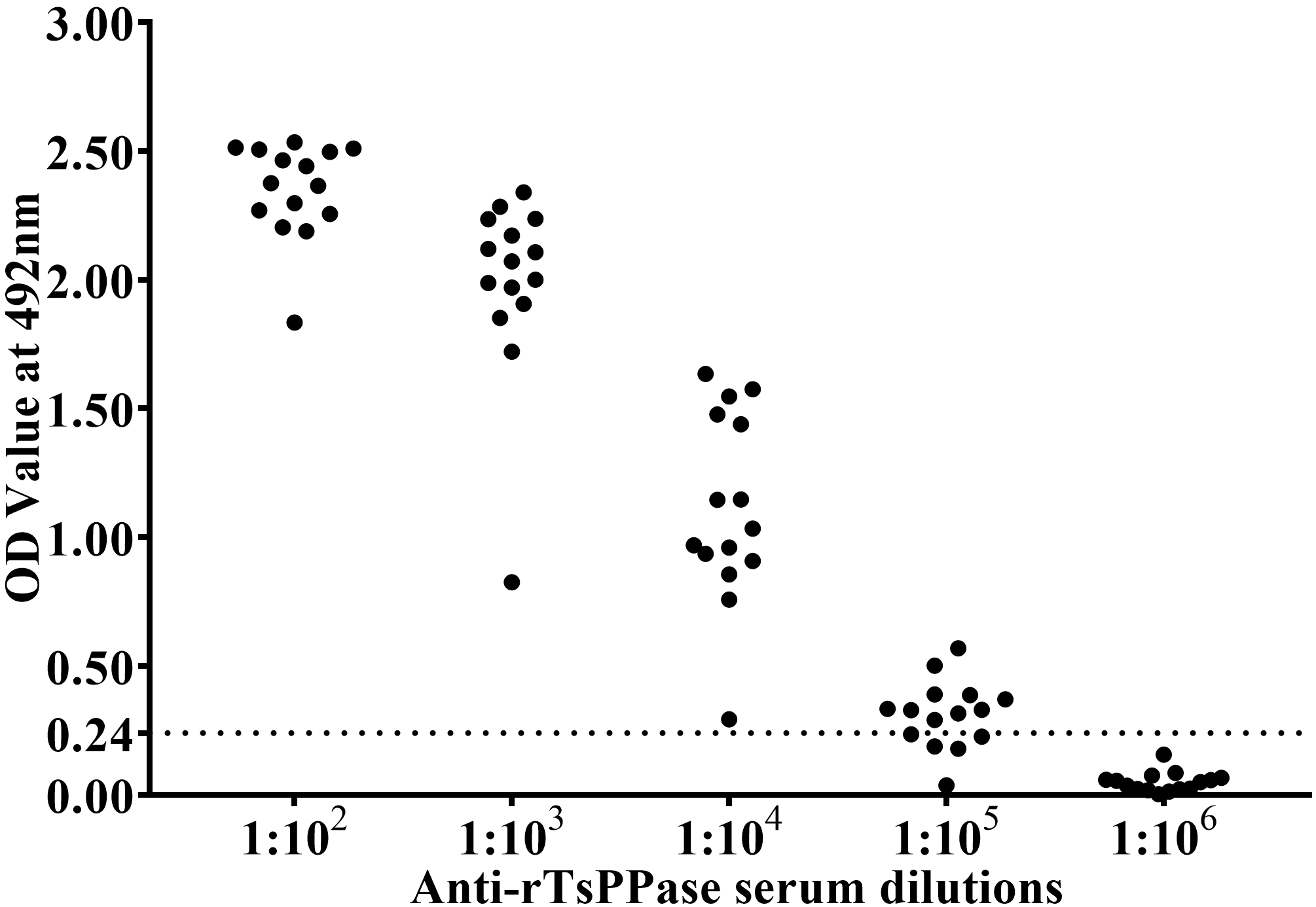


**Additional file 1. Serum anti-rTsPPase IgG titers measured by ELISA with rTsPPase as coating antigen.** Forty normal murine serum samples (1:100 dilutions) were measured as negative controls. The cut-off value (0.241) was showed as a dotted line.
