# Supplementary material for: Biological properties and roles of a Trichinella spiralis inorganic pyrophosphatase in molting and developmental process of intestinal larval stages
Source: Vet Res. 2021 Jan 7;52:6. doi: 10.1186/s13567-020-00877-8 (PMC7791673; doi:10.1186/s13567-020-00877-8)
Supplement: Supplementary file 2 — Additional file 2. IFA of T. spiralis-infected mouse muscle tissue cross sections. Immunostaining was observed at muscle larvae of T. spiralis-infected murine muscle tissue section by IFA using anti-rTsPPase serum, it was primarily localized at stichosome of the larvae. Muscle sections recognized by infection serum as a positive control, and normal serum as the negative control. The nuclei of muscle cells were stained blue by DAPI. Scale bar: 100 μm. [file 13567_2020_877_MOESM2_ESM.docx]

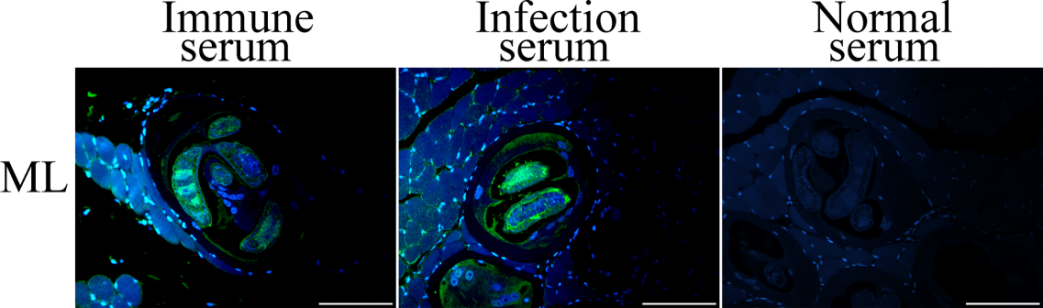


**Additional file 2**. **IFA of *T. spiralis*-infected mouse muscle tissue cross sections.** Immunostaining was observed at muscle larvae of *T. spiralis*-infected murine muscle tissue section by IFA using anti-rTsPPase serum, it was primarily localized at stichosome of the larvae. Muscle sections recognized by infection serum as a positive control, and normal serum as the negative control. The nuclei of muscle cells were stained blue by DAPI. Scale bar: 100 μm.
